# Unmasking EGPA with near fatal diffuse alveolar haemorrhage in severe eosinophilic asthma under treatment with benralizumab: a case report

**DOI:** 10.1186/s12890-026-04106-4

**Published:** 2026-01-10

**Authors:** Nora Drick, Till Frederik Kaireit, Jannik Ruwisch, Sven Schallhorn, Johann Bauersachs, Heiko Schenk, Marius M. Hoeper, Torsten Witte, Benjamin Seeliger

**Affiliations:** 1 Department of Respiratory Medicine and Infectious Diseases, Hannover Medical School, Carl-Neuberg-Str 1, 30625 Hannover, Germany; 2 Biomedical Research in Endstage and Obstructive Lung Disease Hannover (BREATH), member of the German Center for Lung Research (DZL), Hannover, Germany; 3 Institute of Diagnostic and Interventional Radiology, Hannover Medical School, Hannover, Germany; 4 Department of Cardiology and Angiology, Hannover Medical School, Hannover, Germany; 5 Department of Nephrology and Hypertension, Hannover Medical School, Hannover, Germany; 6 Department of Rheumatology and Immunology, Hannover Medical School, Hannover, Germany

**Keywords:** Severe eosinophilic asthma, EGPA, Myocardial involvement, Case report

## Abstract

**Background:**

Eosinophils are key factors to the pathogenesis of severe eosinophilic asthma and eosinophilic granulomatosis with polyangiitis (EGPA). Monoclonal antibodies targeting the interleukin-5 (IL-5) pathway (mepolizumab and benralizumab) often lead to rapid symptom control and enable tapering of oral corticosteroids (OCS) in many patients.

**Case presentation:**

We present the case of a 51-year-old male patient with severe eosinophilic asthma, peripheral blood eosinophilia, and ear, nose, and throat (ENT) involvement, treated with benralizumab (30 mg every 8 weeks) and oral corticosteroids. During tapering of corticosteroids, the patient developed diffuse alveolar haemorrhage as a manifestation of overt vasculitis. Subsequently, elevated troponin T levels were detected, and further diagnostic work-up revealed both myocardial involvement consistent with EGPA and an acute myocardial infarction due to occlusion of the left anterior descending (LAD) artery.

**Conclusion:**

Central immunopathogenic pathways involved in vasculitis are not targeted by IL-5 antibodies and vasculitic manifestations may relapse or even newly emerge despite ongoing biological therapy. Elevated troponin levels in EGPA patients should only be attributed to EGPA once other potential causes of myocardial injury are ruled out.

## Background

The introduction of anti-eosinophilic agents has revolutionized the treatment of severe eosinophilic asthma (SEA), rendering remission an achievable goal even in patients with previously uncontrolled disease. A few patients with SEA may subsequently develop vasculitic symptoms, leading to a diagnosis of Eosinophilic Granulomatosis with Polyangiitis (EGPA), an anti-neutrophil cytoplasmic antibody (ANCA)-associated vasculitis, characterized by asthma, blood and/or tissue hypereosinophilia, and ear, nose, and throat (ENT) involvement [1]. The pathogenesis of EGPA is characterized by a dualistic disease process. An initial eosinophil-driven inflammatory phase, associated with asthma and tissue eosinophilia, is followed or accompanied by an ANCA-associated vasculitic phase affecting small—to medium-sized vessels. These two components may occur sequentially or overlap, contributing to the heterogeneous clinical presentation of EGPA [2]. Eosinophils are central in the pathogenesis of EGPA and two monoclonal antibodies (mepolizumab and benralizumab) blocking the interleukin (IL) 5 pathway have been approved for remission maintenance and treatment of relapsing EGPA [3], demonstrating rapid symptom relief in a substantial number of patients and often allowing for a reduction in OCS dosages. We report the case of a patient receiving benralizumab and OCS for SEA with blood-eosinophilia and ENT involvement, in whom tapering of OCS lead to overt vasculitis.

## Case presentation

**Fig. 1:**
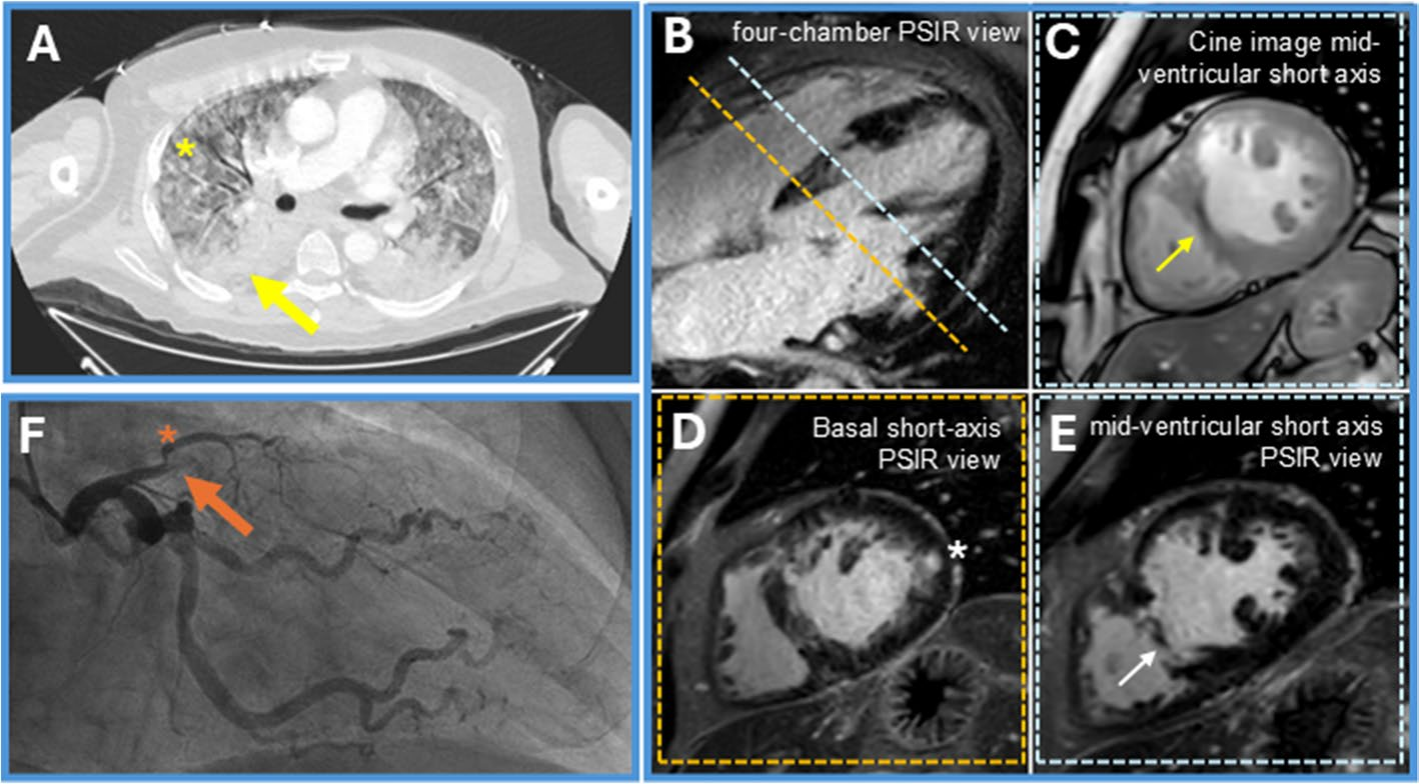
**A** Computed tomography of the chest showing patchy ground glass opacities (asterixis) and basal consolidation (arrow), consistent with diffuse alveolar haemorrhage. **B-E** Cardiac MRI. **B** Late gadolinium enhancement (LGE) image in a four-chamber PSIR view shows both patchy mid-myocardial enhancement and subendocardial to transmural enhancement, illustrating the coexistence of different myocardial injury patterns. **C** Cine image (steady-state free precession) of a mid-ventricular short-axis slice showing thinning of the interventricular septum along with a subendocardial hypo-enhancement in the same region. **D** Basal short-axis PSIR view. An exemplary mid-myocardial LGE in the lateral wall is marked by an asterisk. **E** Corresponding PSIR image reveals an older myocardial scar in the mid-septal region (arrow) but also extensive predominantly subendocardial to transmural enhancement in the anterior and septal walls. These imaging findings are consistent with a coexistence of an old and a more recent infarction along with myocardial involvement in eosinophilic granulomatosis with polyangiitis. Imaging demonstrates both chronic scar tissue and active myocardial injury. T1 mapping and T2-weighted sequences (not shown) confirmed myocardial edema and an elevated extracellular volume fraction (ECV) of 37%. **F** Coronary angiography demonstrating complete occlusion of the left anterior descending artery (orange arrow) after branching of ramus diagonalis (RD, orange asterisk)

A 51-year-old male patient with a history of severe eosinophilic asthma, chronic rhinosinusitis with nasal polyps, and long-term oral corticosteroid (OCS) therapy presented to our outpatient clinic for evaluation of severe asthma. The patient was a never-smoker; known cardiovascular risk factors included obesity and arterial hypertension. Historical laboratory values showed eosinophilia of 1520 cells/µL. Six months prior to presentation, the patient was started on mepolizumab, but no adequate clinical response was achieved. In the presence of persistent exacerbations and failure to taper OCS, treatment was switched to benralizumab (30 mg administered every 4 weeks for the first 3 doses, then every 8 weeks).Owing to a rapid improvement in symptoms and absence of exacerbations, OCS were gradually tapered over a period of 17 months from an initial dose of 40 mg to 5 mg daily, while peripheral blood eosinophil counts remained below 100 cells/μL.While on 5 mg prednisolone daily, the patient developed a productive cough and received antibiotics without improvement. Subsequently, haemoptysis occurred but he did not seek medical attention. Fourteen days after completion of the antibiotic therapy, he experienced major haemoptysis while driving to the doctor’s office, which led to on-site intubation due to respiratory failure and admission to the intensive care unit (ICU). At time of ICU admission, his P_a_O2/F_i_O2 ratio was 140. A chest computed tomography scan revealed diffuse alveolar infiltrates (shown in Fig. 1A), and bronchoscopy confirmed diffuse alveolar haemorrhage. On admission, eosinophil count was 0 cells/µL and myeloperoxidase/ANCA titres were negative. The alveolar haemorrhage was interpreted as a surrogate marker of vasculitis, and in the context of all clinical and diagnostic findings, a diagnosis of eosinophilic granulomatosis with polyangiitis (EGPA) was established. Plasma exchange, intravenous methylprednisolone and intravenous cyclophosphamide at an absolute dose of 1025 mg (500 mg/m^2^) were initiated, leading to rapid respiratory improvement.

While on ICU, a rise in troponin T to 1706 ng/L was noted, and echocardiography showed apical hypokinesia, raising suspicion of EGPA-related cardiac involvement. Cardiac magnetic resonance imaging (MRI) revealed patchy mid-myocardial enhancement and subendocardial to transmural enhancement, suggesting the coexistence of different myocardial injury patterns with a reduced left ventricular ejection fraction of 38% (shown in Fig. 1B-E). With rising troponin despite treatment, coronary angiography was performed and revealed complete occlusion of the proximal left anterior descending (LAD) artery with collateral vessel formation (shown in Fig. 1F). Aortocoronary bypass operation was scheduled and performed 3 months later.

**Fig. 2:**
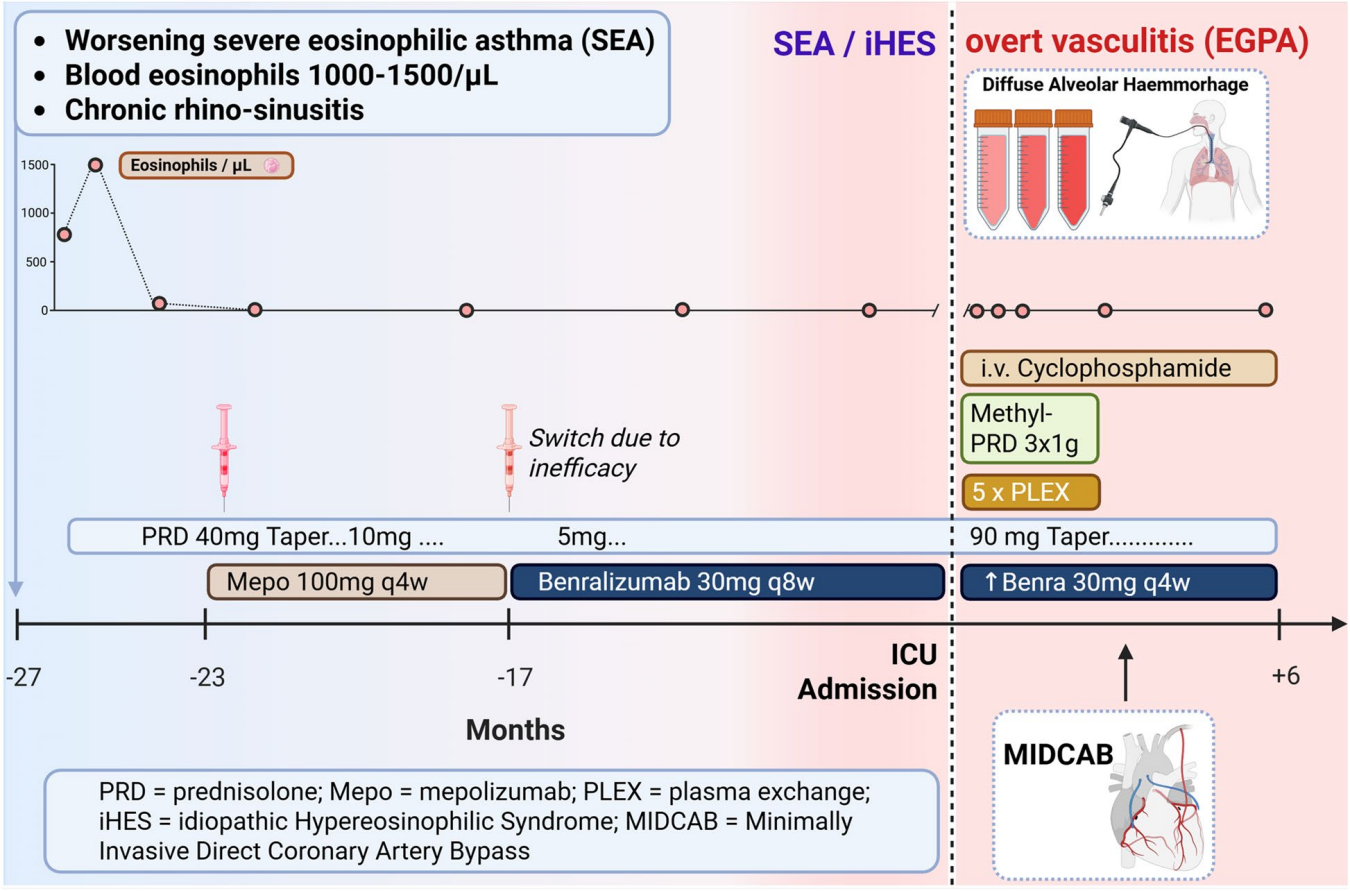
Schematic clinical course and treatment. Blue to red colour fade represents onset of vasculitic (red) features over time

Thereafter, induction therapy with cyclophosphamide was continued at an absolute dose of 1586 mg (750 mg/m^2^) followed by 1000 mg/m^2^ every 4 weeks. OCS were tapered according to the high-dose PEXIVAS protocol, starting at 1 mg/kg body weight of prednisolone with 10 mg reductions every two weeks, followed by a maintenance dose of 5 mg daily [4]. Dosing intervals of benralizumab were shortened to every 4 weeks (q4w). Induction therapy was conducted over 6 months. The patient achieved complete clinical remission. At the first outpatient follow-up visit, the Birmingham Vasculitis Activity Score (BVAS) was 0, compared with 9 at the time of the initial admission to the intensive care unit and 15 during the period of cardiac involvement. The clinical course and treatment are depicted in Fig. 2.

## Discussion and conclusions

This case illustrates new onset vasculitis, presenting as diffuse alveolar haemorrhage, in a patient treated with benralizumab for SEA, concomitant with a gradual reduction in OCS dosage. Since the approval of benralizumab for EGPA in 2024, a second monoclonal antibody targeting the IL-5 pathway is available for remission maintenance and treatment of relapsing EGPA. Both mepolizumab and benralizumab interfere with eosinophilic inflammation and rapidly resolve blood-eosinophilia [5]. As SEA and ENT manifestations are leading symptoms of EGPA, both induced by eosinophilic inflammation, many patients experience significant symptom reduction under anti-eosinophilic treatment, often enabling dose reduction of OCS [6]. Benralizumab has demonstrated sustained long-term efficacy in patients with EGPA, enabling OCS-free remission in the majority of patients [7]. Importantly, since vasculitis is possibly mediated by Th1/Th17 driven pathways [8] and not addressed by anti-IL-5 therapy, relapse or de-novo vasculitis may occur when tapering OCS and reducing immunosuppressant effects. As glucocorticoids suppress key pro-inflammatory pathways, including Th1- and Th17-mediated cytokine production, dose reduction may lead to reactivation of these T-cell subsets and consecutive increased secretion of pro-inflammatory cytokines such as IFN-γ and IL-17, with a subsequent emergence of vasculitic symptoms [9]. Accordingly, vasculitis flares have been observed in EGPA-patients with a history of histologically confirmed vasculitis while treated with benralizumab [6], particularly in ANCA-positive patients during corticosteroid tapering. Data are very scarce, but existing studies primarily describe relapses of overall disease activity rather than newly onset vasculitis [10]. On this account, it is of utmost importance that OCS are tapered gradually and that patients are carefully monitored for vasculitic symptoms during the tapering process. Such symptoms may include neuropathy, purpura, haemoptysis or ENT involvement. Regular assessment of eosinophil counts, renal function, and ANCA status may help detect early vasculitic manifestations and guide timely treatment adjustments. Notably, a negative ANCA status does not preclude the diagnosis of EGPA as approximately 60% of EGPA-patients are ANCA-negative [3]. A recent study screening over 500 patients with severe asthma reported that an EGPA diagnosis was highly probable in nearly 4% of cases [11].

The emergence of vasculitis during anti-IL-5 therapy highlights the dual pathophysiology of EGPA, with both eosinophil- and vasculitic mechanisms. While the approved biologics target eosinophilic inflammation, vasculitic features may require immunosuppressant treatment regiments. One recently published case report supports a dual-targeted approach in selected patients with vasculitic manifestations [12], underscoring the need for individualized, mechanism-based management. Evidence-based guidelines recommend remission induction therapy with cyclophosphamide or rituximab, in addition to high-dose oral corticosteroids, in patients with severe or life-threatening EGPA [3]. In our case, treatment with cyclophosphamide was initiated, and due to the good response in terms of ENT and asthma symptom control, benralizumab therapy was continued, with the dosage adjusted to the approved regimen for EGPA.

Both diffuse alveolar haemorrhage and myocardial involvement are considered vasculitis surrogates [13]. In our case, cardiac MRI indicated cardiac involvement of EGPA and consequently the initial increase in cardiac troponin was therefore attributed exclusively to vasculitic involvement. Following work-up, both EGPA cardiac involvement and occlusive coronary artery disease were co-existent with significant therapy implications. Evidently, differential diagnoses for troponin elevation must be ruled out. This consideration is especially important in patients with cardiovascular risk factors as well as in those who fail to show an adequate therapeutic response. Evaluation of troponin elevation in EGPA patients should always consider cardiovascular risk factors and correlate troponin levels with clinical symptoms. Cardiac imaging should include both transthoracic echocardiography and MRI, and coronary angiography should be performed if troponin elevation persists or an ischemic etiology cannot be excluded. A study screening asymptomatic EGPA patients detected early signs of cardiac involvement in 45% of cases, supporting the value of early cardiac assessment in this population [14]).

In summary, in this case benralizumab for treatment of SEA enabled OCS reduction, which was followed by the onset of EGPA-associated vasculitis. Central immunopathogenic pathways involved in vasculitis are not targeted by IL-5 antibodies and vasculitic manifestations may reappear or even newly emerge despite ongoing biological therapy. Notably, the patient also presented with coexistent myocardial infarction due to LAD occlusion and myocardial involvement of EGPA. This case illustrates the need for vigilant monitoring for vasculitic and cardiac complications in patients treated with anti-eosinophilic biologicals. Elevated troponin levels in EGPA patients should not automatically be attributed to EGPA as differential causes of myocardial injury have to be ruled out, especially in patients with underlying cardiac risk factors. Clinicians should remain vigilant for vasculitis symptoms and the potential new onset of vasculitis during biologic therapy, particularly in patients with severe asthma undergoing oral corticosteroid tapering.

## Data Availability

All data associated with this paper are available, and data not presented in the manuscript can be accessed from the corresponding author upon reasonable request.

## Abbreviations

ANCA: Anti-neutrophil cytoplasmic antibody
EGPA: Eosinophilic granulomatosis with polyangiitis
ENT: Ear, nose, and throat
ICU: Intensive care unit
IL: Interleukin
LAD: Left anterior descending
MRI: Magnetic resonance imaging
OCS: Oral corticosteroids
Q4w: Every 4 weeks
Q8w: Every 8 weeks
SEA: Severe eosinophilic asthma
